# The influence of 'significant others' on persistent back pain and work participation: A qualitative exploration of illness perceptions

**DOI:** 10.1186/1471-2474-12-236

**Published:** 2011-10-14

**Authors:** Serena McCluskey, Joanna Brooks, Nigel King, Kim Burton

**Affiliations:** 1 Centre for Health & Social Care Research, University of Huddersfield, Queensgate, Huddersfield, HD1 3DH, UK; 2 Centre for Applied Psychological Research, University of Huddersfield, UK

## Abstract

**Background:**

Individual illness perceptions have been highlighted as important influences on clinical outcomes for back pain. However, the illness perceptions of 'significant others' (spouse/partner/close family member) are rarely explored, particularly in relation to persistent back pain and work participation. The aim of this study was to initiate qualitative research in this area in order to further understand these wider influences on outcome.

**Methods:**

Semi-structured interviews based on the chronic pain version of the Illness Perceptions Questionnaire-Revised were conducted with a convenience sample of UK disability benefit claimants, along with their significant others (n = 5 dyads). Data were analysed using template analysis.

**Results:**

Significant others shared, and perhaps further reinforced, claimants' unhelpful illness beliefs including fear of pain/re-injury associated with certain types of work and activity, and pessimism about the likelihood of return to work. In some cases, significant others appeared more resigned to the permanence and negative inevitable consequences of the claimant's back pain condition on work participation, and were more sceptical about the availability of suitable work and sympathy from employers. In their pursuit of authenticity, claimants were keen to stress their desire to work whilst emphasising how the severity and physical limitations of their condition prevented them from doing so. In this vein, and seemingly based on their perceptions of what makes a 'good' significant other, significant others acted as a 'witness to pain', supporting claimants' self-limiting behaviour and statements of incapacity, often responding with empathy and assistance. The beliefs and responses of significant others may also have been influenced by their own experience of chronic illness, thus participants lives were often intertwined and defined by illness.

**Conclusions:**

The findings from this exploratory study reveal how others and wider social circumstances might contribute both to the propensity of persistent back pain and to its consequences. This is an area that has received little attention to date, and wider support of these findings may usefully inform the design of future intervention programmes aimed at restoring work participation.

## Background

Musculoskeletal conditions in the workplace account for 9.5 million days of work absence in the UK, and persistent back pain accounts for around 20 per cent of claims for long-term state benefit [1,2]. The costs of reduced work capacity due to persistent back pain greatly outweigh its direct medical costs [3], and it is now widely accepted that remaining in work, or returning to work as soon as possible is generally beneficial in order to avoid the adverse physical, mental and social effects associated with long-term worklessness [4,5]. Extensive research has established that psychosocial risk factors are important influences on recovery and work participation, but the fact remains that the majority of individuals who have been on prolonged sick-leave due to persistent back pain will not return to any form of work in the foreseeable future [6]. It has recently been proposed that illness perceptions may emerge as important influences on outcome for those with persistent back pain [7], and that more qualitative research in this area may provide a better understanding of psychosocial obstacles to work participation [7,8].

Illness perceptions are derived from the self-regulatory model (SRM) of health behaviour [9], which provides a framework for understanding the processes by which an individual's own implicit, common-sense beliefs about illness are associated with behavioural responses employed to manage outcomes. Specifically, it has been demonstrated that illness perceptions have a significant association with clinical outcomes for back pain over a 6-month period [10,11], and results from a recent systematic review suggest that illness perceptions may play an important role in mediating between illness and work outcomes [12]. Whilst the importance of individual illness perceptions continues to be documented, there is less understanding of the illness representations of 'significant others' (spouse/partner/close family member), particularly in relation to persistent back pain and work participation.

Several studies have proposed that significant others have an important influence on an individual's pain, illness behaviour, and pain-related dysfunction, and that they may be particularly salient sources of discriminative cues, punishment or reinforcement for pain behaviours [13,14]. Cognitive-behavioural theory suggests that the pain-related beliefs and cognitions of close others affect the development, maintenance, and management of pain and distress [15], and it has been found that spousal pain beliefs about disability, emotion, control and medication are significantly correlated with partners' pain severity and other indicators of pain adjustment [16]. More specifically, recent research exploring general beliefs and attitudes about health and work in those currently claiming disability benefit in the UK included spousal interviews, and concluded that family had a role to play in terms of being able to support and ideally facilitate behaviour change, and that the concept of mutual support and encouragement is very important in promoting work participation [17,18]. Thus, an exploration of the illness perceptions of individuals with disabling back pain and those of their significant others may offer a further insight into these influences.

## Method

### Study design

Individual semi-structured interviews based on the chronic pain version of the revised Illness Perceptions Questionnaire (IPQ-R) [19] were conducted. The IPQ-R provides a quantitative measure of illness perceptions for a number of conditions, but this method was chosen in an attempt to elucidate more in-depth data about the experience of persistent and disabling back pain, allowing participants to fully elaborate on each question. Epistemologically, the study falls within what Hammersley refers to as a "subtle realist" approach [20]. This position recognises that research is never independent of the perspective of the particular researcher(s) involved. However, unlike radical relativist approaches such as social constructionism, it does advocate the existence of a reality outside of, and knowable to, the researcher. Relevant permissions for the study were obtained from NHS Research Ethics (reference no: 10/H1014/19).

### Participants

A convenience sample of disability benefit claimants were recruited from the Lancashire Condition Management Programme (CMP) in the North-West of England. Only those claimants who reported non-specific back pain as their main condition were identified and invited to participate in this study by their case manager. Claimants also had to be at the point of entry into CMP and have a 'significant other' in order to be included in the study. CMPs were conceived as part of the UK government's Pathways to Work Initiative [21] and provide support to those claimants with mild to moderate mental health, musculoskeletal or cardio-respiratory problems who wish to make steps to return to work. CMP personnel include clinicians from a variety of professional backgrounds including occupational therapy, physical therapy and nursing who provide active case-management with individually tailored programmes of 16 weeks average duration.

Participants were given full study information sheets and written informed consent was obtained. The mean age of claimant participants was 41.0 years (ranging from 29 to 54 years), and the mean age of significant other participants was 40.2 years (ranging from 21 to 62 years). With the exception of one claimant, none had continued their education past high school, and all had previously worked in manual occupations. All but one of the claimants were male, and all significant others were female. Three of the participant dyads were in spousal relationships, and two were parent/child dyads. All participants described themselves as belonging to the 'White British' ethnic group.

### Interviews

Claimants and significant others were interviewed separately at a time convenient to them in their own homes, with the interviews lasting around one hour. The interview schedule included the same or similar questions to those making up the nine subscales of the IPQ-R: illness identity; timeline (acute/chronic); timeline (cyclical); consequences of illness; personal control over illness; treatment control over illness; emotional representations; illness coherence; and beliefs about causality. Interviews were digitally recorded and transcribed in full.

### Data analysis

Interview transcripts were analysed using template analysis - a method which provides a systematic technique for categorising qualitative data thematically, and which has been used previously in both healthcare [22] and occupational research [23]. Template analysis was chosen because it allows a-priori themes to be used to help develop an initial version of the coding template - in this case, the nine subscales of the IPQ-R. An initial template was constructed using the significant other interview data, as the perceptions of these participants were the central focus of the study. Claimant interview data were then mapped onto the initial template, modifying it further until all relevant data were coded satisfactorily. The process was concluded by applying the final version of the template to the data as a whole, and a-priori themes were redefined or discarded if they did not prove to be helpful in capturing key meanings in the data.

The final coding template was comprised of six IPQ-R constructs, compared with the nine questionnaire subscales (see McCluskey et al for further detail) [24]. Two additional top-level themes were also defined in the course of the analysis relating to: establishing the authenticity of incapacity (labelled as 'claimant as genuine'); and the role of a significant other (labelled as 'influence of/impact on significant others'). For the purposes of the present article, only those themes which incorporate data most clearly related to work participation will be presented. These themes were labelled: 'beliefs about causality'; 'consequences of illness; 'claimant as genuine'; and 'influence of/impact on significant other'. Verbatim extracts from the interview data are presented in order to illustrate these four themes, and pseudonyms have been used throughout.

## Results

### 1. Beliefs about causality

All claimants reported work as the initial cause of their back pain condition, and most also perceived previous work/certain types of work (manual/heavy/repetitive) as a 'trigger' for subsequent episodes and therefore not conducive to return to work. These beliefs were also shared by significant others:

"I didn't have any problem with it up until going into that job and that's why I've put it down to doing those things....if I'm in a job where I'm sitting down all day or standing or whatever at a machine all day then it's going to go, it's going to continue to go"[Alistair - claimant]

"It's probably something that he carried in work that hurt his back" [Paula - significant other]

----------------------------------------------------

"Well many moons ago I would say yes I'll take on manual jobs, but there again carrying slate up on the roof with a bad back isn't an option unfortunately" [Adrian - claimant]

"He was doing a job which involves lifting a lot of things" [Nadia - significant other]

----------------------------------------------------

"All the time I've found it comes on more the harder work I'm doing....it's the heavy building I think ... and certain jobs" [Michael - claimant]

"Manual work he really struggles with" [Sally - significant other]

----------------------------------------------------

Interestingly, participants attributed the persistence of the back pain condition to physical work-environment risk factors. However, evidence suggests that such risk factors only account for between 10 and 30 per-cent of long-term sickness absence due to chronic back pain, and that the rest can be attributed to psychosocial risk factors [25]. Indeed, the influence of psychosocial risk factors such as psychological distress, fear-avoidance, catastrophizing, unhelpful pain beliefs and behaviour, job dissatisfaction and perceived lack of social support in the workplace on claimants' work incapacity became more apparent during further analysis of the interview data, in particular revealed within the 'consequences of illness' theme.

### 2. Consequences of illness

Responses suggested that claimants had become self-limiting and fearful of work activity, and were pessimistic about the likelihood of a return to work. Significant others seemed to share and perhaps further reinforce these unhelpful beliefs, and in some cases, were more resigned to the permanence and inevitable negative consequences of the back pain condition on work participation. Significant others in particular were sceptical about the availability of suitable work and sympathy from employers:

"What's important is that I'm not sat down or stood still or something like day after day because it'll stop me from walking, which will stop me from working" [Alistair - claimant]

"And, as I say to him, who's going to hire you? With a backache, you know......how can he get a job with, you know, his back the way it is, when he can't sit down too long, he can't walk too long, he has to lie down. And who's gonna let him lie down when he's working in the factory, no-one are they?" [Paula - significant other]

----------------------------------------------------

"Well I can't lift anything heavy. I can't bend over properly; I'm limited in my movement about 60%" [Adrian - claimant]

"I know that there's jobs he wants to do out there but he can't because of his back" [Nadia - significant other]

----------------------------------------------------

"I would be looking for an employer that would not mind if I didn't turn up for several weeks at a time when I have a flare up, which I was suffering with last year for 24 weeks out of the year...it's bringing my brain to work but not necessarily my body" [Helen - claimant]

"If she's got the freedom to stand up and work or sit down for 10 minutes, if she can jiggle herself around then she can work" [Jill - significant other]

----------------------------------------------------

"I don't ever think that I'll be able to do anything in the construction trade" [Michael - claimant]

"He can't work because he's got so much back pain" [Sally - significant other]

----------------------------------------------------

"I can't imagine me going back to work" [Roger - claimant]

"If he does go back to work what's he even going to be able to do?" [Lydia - significant other]

----------------------------------------------------

It has previously been suggested that welfare systems may promote the problem of disability by rewarding sickness absence [26], and therefore perhaps claimants may have become increasingly self-limiting and fearful of work activity in order to fulfil their 'disabled role' and not appear fraudulent. This 'pursuit of authenticity' became more apparent during further analysis of interview data, leading to the emergence of a new theme labelled 'claimant as genuine'.

#### Claimant as 'genuine'

Perhaps so as not to appear fraudulent, claimants felt the need to stress their desire to work, but emphasised how the severity and physical limitations of the condition prevented them from doing so. Here, significant others seemed to act as a 'witness to pain', validating the claimants' statements of incapacity and self-limiting behaviour:

"The only time I used to go out last year was when I was looking for work, I used to go out every day looking for work...If I didn't have a back problem I'd be working" [Alistair - claimant]

"But you can see, you can see it in his face, you know, when he's walking....he walks around for about ten, twenty minutes and then he has to go back upstairs and lie on his bed" [Paula - significant other]

----------------------------------------------------

"I've always worked since I came out of school ..... well I carried on working in the evenings when I was at school and not being able to work has crippled me. I had three jobs at one time; I was working in three jobs, and to go from three jobs to nothing..." [Adrian - claimant]

"I can probably tell when I can see the way he walks if he's sore or not....I could see how much pain he was in ... even sitting down for more than half-an-hour" [Nadia - significant other]

----------------------------------------------------

"I'm not a lazy-bones, I don't wanna be on the sick, I wanna work" [Michael - claimant]

"He tries to like, just get through it and carry on .... he has tried, so it's not like he's not been trying" [Sally - significant other]

----------------------------------------------------

"It's not affecting me mentally if that's what you think .... there's nothing wrong with my head and my brain, it's my physical body" [Roger - claimant]

"I've seen, you know living with somebody who's in a lot of pain ..... he really can't do anything" [Lydia - significant other]

----------------------------------------------------

Significant others were keen to further demonstrate their dedication and support of the claimants, presenting themselves as 'good' significant others. These findings are illustrated further in another additional theme described below.

#### Being a 'good' significant other

An important finding in this study was that *all *significant others also had long-term health conditions, with some being disability benefit claimants themselves. This appears to have led to a shared understanding and high degree of empathy with claimants:

"I think we sort of get sympathy pains for each other" [Nadia - significant other]

----------------------------------------------------

"Maybe we're an odd household because we're both ill that that makes us more understanding of each other" [Jill -significant other]

----------------------------------------------------

"I think if I didn't get this [back pain] I'd be more, 'get up, you know, stop whining and just carry on', but no, recently he's not been able to, to carry on, and I sympathise with him a lot" [Sally - significant other]

----------------------------------------------------

Seemingly as a further attempt to display their dedication to the claimant, significant others illustrated how they 'helped' the claimant in their everyday lives, reporting high levels of routine dependency:

"I just help him, run up and down stairs, run up and down stairs when he wants....if he wants something he can ask me and I'll do it for him" [Paula - significant other]

----------------------------------------------------

"I cook for him so he doesn't have to stand and cook, if he has problems getting in and out of bed I'll try and help him as much as I can,... pretty much anything that he can't do I'll try and do....I'm willing to do it" [Nadia -significant other]

----------------------------------------------------

"She needs a lot of physical support and stuff while it's going on....she can't really do anything for herself when she's that bad she just has to lay in bed and be waited on" [Jill - significant other]

----------------------------------------------------

"I come and cook and keep things tidy" [Sally - significant other]

----------------------------------------------------

## Discussion

The findings from this exploratory study reveal novel and interesting insights about the beliefs and behaviours of significant others in relation to disabling back pain, and how these may act as obstacles to work participation. Significant others shared, and perhaps further reinforced, claimants' unhelpful beliefs including fear of pain/re-injury associated with certain types of work and activity, and pessimism about the likelihood of return to work. In some cases, significant others appeared more resigned to the permanence and negative inevitable consequences of the claimant's back pain condition on work participation, and were more sceptical about the availability of suitable work and sympathy from employers. In their pursuit of authenticity, claimants were keen to stress their desire to work whilst emphasising how the severity and physical limitations of their condition prevented them from doing so. In this vein, and seemingly based on their perceptions of what makes a 'good' significant other, significant others acted as a 'witness to pain', supporting claimants' self-limiting behaviour and statements of incapacity, often responding with empathy and assistance. The beliefs and responses of significant others may also have been influenced by their own experience of chronic illness, thus participants lives were often intertwined and defined by illness.

Unfortunately, the small sample size in this exploratory study meant that it was not possible to establish whether the illness perceptions of significant others had a direct association with claimant illness perceptions and subsequent behavioural responses and/or outcomes. Relatively little research investigating the influence of significant others' illness perceptions on the illness beliefs of partners has been conducted [27], and more research of this nature with larger samples is required. The small sample size also means that other possible influences on claimant beliefs, such as socio-demographic/economic characteristics cannot be further explored, but which we believe may be important. Nevertheless, many findings in this study are supported by those documented in other studies of chronic back pain, those investigating the influence of significant others on illness, and qualitative research on work participation, all with wider sample heterogeneity.

For example, the desire to be seen as genuine is a common finding in populations experiencing persistent and disabling back pain, probably because it is likely that the cause remains medically unexplained, as is the case for 90% of sufferers [28]. It has been shown that the avoidance of cultural stereotypes associated with unexplained back pain (such as 'malingerer') becomes very important and individuals feel the need to establish their credibility, proving their pain is real [29,30]. In addition, the associations that participants made between type of work (i.e. manual/heavy/repetitive) and inflexible or unsympathetic employers have been reported in other research carried out with disability benefit claimants [31], and it should be acknowledged that the ability to re-train or obtain further educational qualifications in order to move into a more suitable occupation is often out of reach for disability benefit claimants due to financial constraints, existing educational level, and limitations posed by ill-health. This highlights the difficulties faced by certain groups in the population in maintaining work participation, and illustrates how sickness absence is mediated by wider social factors [32]. This is supported by the findings from a systematic review of the qualitative literature on return to work after injury which suggest that return-to-work extends beyond concerns about managing physical function to the complexities related to beliefs, roles and perceptions of those involved [33].

It is acknowledged that qualitative research has its limitations, with criticisms usually focused on issues related to validity and reliability. However, the theoretical model on which this study was based (the SRM) has been successfully applied in many studies investigating various health conditions across a number of settings, and there are several examples in the published literature where qualitative research methods have been used to investigate and elaborate upon its components [34-36]. More specifically, a semi-structured interview based on the IPQ-R was recently used in a study of patients with sciatica [37]. It should also be stressed that this study was conceived in response to a call for more qualitative research in this area, and therefore the chosen methodology and the inclusion of significant others helped initiate a wider investigation of psychosocial obstacles to work participation. Further research of this nature is currently underway, and it is hoped that findings will add weight to those of the current study.

Future research may also need to incorporate an examination of the perceived role expectations within relationships (i.e. between parent and child and between spouses) and between genders (for example, all significant others in this study were female), and also the influence that others' own health problems may exert. Findings from the current study appear to support other research which suggests that pain-related empathy is seen by many partners as an essential ingredient of relationship quality, being viewed by many as an important requisite for meeting the patient's support needs [38]. However, the difference between support and solicitous behaviour (responding to pain with expressions of empathy and assistance) may not be clearly understood and unfortunately, such behaviour is linked to poorer functional outcomes, greater pain behaviours and reports of greater pain intensity in patients with chronic pain [39-42]. Further investigation of the influence of significant others (who could also be defined as healthcare professionals, employers and those in the welfare system) appears to be a promising area of research and may provide a better understanding of mechanisms that facilitate work retention and effective vocational rehabilitation.

## Conclusions

Findings from this study highlight some of the everyday life experiences of those with persistent and disabling back pain, and help illustrate the complexity that can be involved in work participation for those on long-term sickness absence. What this study importantly suggests is that rather than focus solely on individual risk factors for long-term sick leave, it may be important to understand how others and wider social circumstances might contribute both to the propensity of persistent back pain and to its consequences. This is an area that has received little attention to date, and wider support of these findings may usefully inform the design of future work participation programmes.

## Competing interests

The authors declare that they have no competing interests.

## Authors' contributions

All authors participated in the design and conception of the study. SM & JB conducted the interviews. SM, JB & NK read, coded and analysed the data. KB checked the coding and analysis. SM drafted the manuscript, and all authors read, revised, and approved the final manuscript.

## Pre-publication history

The pre-publication history for this paper can be accessed here:

http://www.biomedcentral.com/1471-2474/12/236/prepub
